# Supplementary figures and images for: Evaluation of Aedes aegypti, Aedes albopictus, and Culex quinquefasciatus Mosquitoes Competence to Oropouche virus Infection
Source: Viruses. 2021 Apr 25;13(5):755. doi: 10.3390/v13050755 (PMC8145018; doi:10.3390/v13050755)

Figure S1

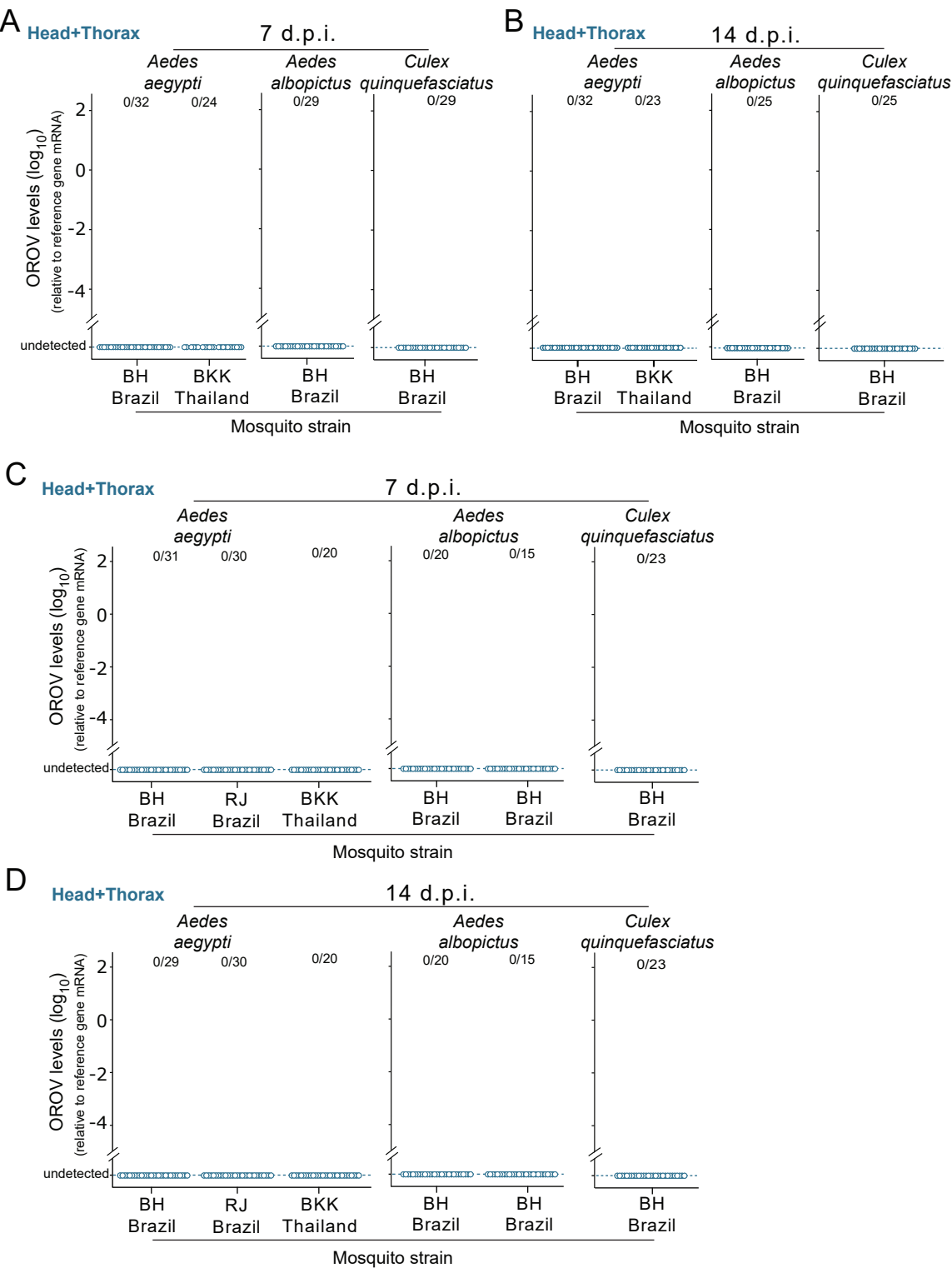

Supplement: Supplementary file 1 [file viruses-13-00755-s001.zip › viruses-1166715-supplementary.pdf]
